# Mechanism and Substrate Recognition of Human Holo ACP Synthase

**DOI:** 10.1016/j.chembiol.2007.10.013

**Published:** 2007-11-26

**Authors:** Gabor Bunkoczi, Saloni Pasta, Anil Joshi, Xiaoqiu Wu, Kathryn L. Kavanagh, Stuart Smith, Udo Oppermann

**Affiliations:** 1 Structural Genomics Consortium, University of Oxford, Oxford OX3 7LD, UK; 2 Children's Hospital Oakland Research Institute, Oakland, CA 94609, USA

**Keywords:** CHEMBIO, PROTEINS

## Abstract

Mammals utilize a single phosphopantetheinyl transferase for the posttranslational modification of at least three different apoproteins: the carrier protein components of cytosolic and mitochondrial fatty acid synthases and the aminoadipate semialdehyde reductase involved in lysine degradation. We determined the crystal structure of the human phosphopantetheinyl transferase, a eukaryotic phosphopantetheinyl transferase characterized, complexed with CoA and Mg^2+^, and in ternary complex with CoA and ACP. The involvement of key residues in ligand binding and catalysis was confirmed by mutagenesis and kinetic analysis. Human phosphopantetheinyl transferase exhibits an α/β fold and 2-fold pseudosymmetry similar to the Sfp phosphopantetheinyl transferase from *Bacillus subtilis*. Although the bound ACP exhibits a typical four-helix structure, its binding is unusual in that it is facilitated predominantly by hydrophobic interactions. A detailed mechanism is proposed describing the substrate binding and catalytic process.

## Introduction

**Figure 1:**
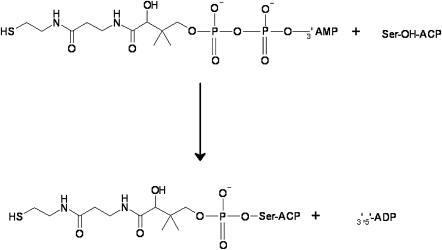
Reaction Catalyzed by Human PPT A conserved Ser-OH nucleophile in the ACP acceptor protein is modified through modification by the (β-) phosphopantetheinyl group of CoA to yield holo-ACP.

The posttranslational modification of a conserved serine residue in carrier proteins with a phosphopantetheinyl moiety transferred from coenzyme A through the enzyme phosphopantetheinyl transferase (PPT) (Figure 1) is vital to all living organisms. The resulting holo-carrier proteins play essential roles as components of fatty acid and polyketide synthases (acyl carrier proteins, ACPs) and nonribosomal peptide synthases (peptidyl carrier proteins, PCPs) [1]. In addition, phosphopantetheinylated carrier proteins are also involved in the metabolism of lysine and tetrahydrofolate [2–4]. In each of these systems, the reactive sulfhydryl of the flexible 20 Å-long phosphopantetheine arm provides a center for the transport of reaction intermediates between the various catalytic components of the pathway.

Whereas microbial genomes contain more than one PPT [5–8], only one PPT has been identified in mammals. This enzyme exhibits a broad substrate specificity and is able to phosphopantetheinylate the ACP components of the cytosolic and the mitochondrial FAS systems, as well as aminoadipate semialdehyde dehydrogenase, the human ortholog to the yeast *lys5* gene [2, 9]. Subcellular fractionation studies indicate that the human PPT is located in the cytosolic compartment, implying that the mitochondrial ACP and aminoadipate semialdehyde dehydrogenase undergo posttranslational modification prior to their import into mitochondria. Molecular cloning and biochemical characterization of human PPT revealed that this cytosolic enzyme is a Mg^2+^-requiring monomeric enzyme that exhibits a broad acceptor substrate specificity [9].

Based on sequence motifs and structural features, phosphopantetheine transferases are classified in three different groups [6]. Group I is represented by bacterial transferases of about 120 residues, acting on the ACP of bacterial FASs with *Escherichia coli* or *Bacillus subtilis* ACPs as prototype members. Structure determination of the group I PPT (designated as holo-ACP synthase, AcpS) from *B. subtilis* and *Streptococcus pneumoniae* revealed a trimeric arrangement of an α/β fold domain [10, 11]. The active site is located in the cleft between the different subunits, resulting in three active sites per molecule. Group II PPTs are exemplified by the Sfp protein from *B. subtilis*, which catalyzes the 4′-phosphopantetheine transfer from CoA to the nonribosomal, PCP domain of bacterial surfactin synthetase [7, 12, 13]. Sfp is a monomer of about 240 residues and exhibits a broader acceptor substrate specificity than group I PPTs. The crystallographic analysis of Sfp [13] revealed a two-domain enzyme with intrinsic pseudo 2-fold symmetry, however, with a similar domain architecture as trimeric AcpS. The 4′-phosphopantetheine donor is bound in a cleft formed by the two domains, and the structure revealed basic principles of Mg^2+^ and CoA binding. Group III PPTs comprise transferases that are an integral part of the heteromeric yeast and fungal FASs [14], which display a different architecture than mammalian FAS systems [15–17]. The group III PPTs appear to act on their apoACP substrates prior to assembly of the megasynthase complex since, in the context of the complete complex, the PPT structure is distorted and incapable of binding CoA [16].

The closest structurally characterized enzyme to human PPT is the group II Sfp enzyme from *B. subtilis* (PDB ID: 1QR0 [13]). Here, we describe the structure determination of apo and substrate complexes and provide biochemical analysis of human PPT, to our knowledge the first higher eukaryotic representative of this class of enzymes. These data suggest a general reaction mechanism for eukaryotic PPT and, as far as we know, furthermore provide for the first time structural evidence that type I and II ACPs distinguish their substrate molecules through distinct recognition mechanisms.

## Results and Discussion

### Overall Structure of Human PPT

**Figure 2:**
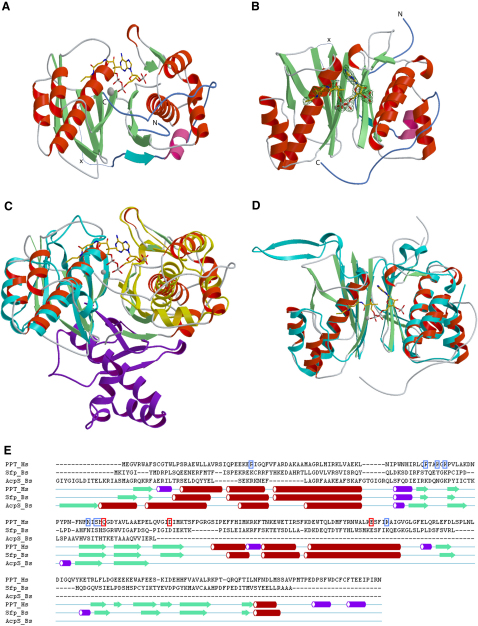
Structural Comparison of the PPT Family (A) Ribbon model of human PPT in complex with CoA and Mg^2+^ revealing the 2-fold pseudosymmetry of group II PPTs. (B) Similar view of human PPT rotated 90° around the y axis, CoA electron density (positive difference map, contoured at 2σ) displayed in blue. N and C termini are highlighted in blue and labeled N and C, respectively. The loop with missing electron density is indicated by x. (C) Overlay between human PPT and bacterial AcpS (trimeric subunits displayed in yellow, cyan, and violet), showing active sites located in the domain clefts both for group I and II PPTs. (D) Overlay of group II PPTs (cyan, *Bacillus subtilis* Sfp; red/green, human PPT). (E) Structural alignment of human PPT, Sfp, and AcpS highlighting conserved motifs and secondary structure elements. CoA binding residues are boxed in light blue, catalytic residues in red. One AcpS molecule is included in the alignment to highlight similar secondary structure features. The structural alignment and the plot were generated with the ICM software package [35], extended strands are depicted in green, alpha helices in red, and 3/10 helices in purple.

Human PPT consists of two nearly identical domains connected by a short linker region. The two halves superimpose on each other with an rmsd of 2.4 Å but are distinguished by two features. First, the N- and C-terminal regions of the protein contain 16 and 42 residue extensions, respectively, that are unique to each half of the molecule. Both extensions are well ordered, and only the terminal four-five residues are indistinguishable from the noise in the electron density maps. Second, although the regions encompassing residues 91–116 in the N-terminal domain and residues 207–239 in the C-terminal domain both fold into a pair of β sheets connected by a loop region, the extension in the C-terminal domain is significantly longer (Figure 2).

The overall fold adopted by human PPT most closely resembles that of the *B. subtilis* Sfp protein with the exception of the N- and C-terminal extensions that are unique to the human enzyme. The C-terminal extension folds back to the N-terminal domain and possibly fixes the relative orientation of the domains so that it is optimal for catalysis. Both the human and *B. subtilis* enzymes contain a pair of positionally equivalent β sheets looped out from the core structure in the C-terminal domain: residues 196–227 in PPT and 160–187 in Sfp. However, in Sfp this region is involved in an extended crystal contact and protrudes from the main body of the protein up to 45 Å. Reuter et al. [13] have speculated that this region may be involved in apoprotein binding.

The functional domains can be defined with the help of the group I PPT AcpS, which consists of a shorter chain that trimerises to form the functionally active protein. Superposition of this structure on the N- and C-terminal domains of human PPT (Figure 2) reveals that N-terminal 13 and the C-terminal 52 residues can be regarded as extensions and that the linker between the N- and C-terminal domain consists of a single residue. Another feature is the disordered loop between residues 248–263, which starts after the superposition of the PPT C-terminal domain and AcpS ends, suggesting that this may act as a flexible linker.

### Structure of the Fatty Acid Synthase ACP Domain and Binding to PPT

ACPs are classified into two distinct groups, type I representing those that are part of the multifunctional polypeptides that constitute the cytosolic animal FAS and modular polyketide synthases and type II that are associated with the freestanding enzyme systems involved in fatty acid and polyketide synthesis in bacteria, plastids, and mitochondria. Although several type II ACP structures have been determined experimentally, only one type I structure has been solved, that of the rat FAS, using NMR [18]. We have now determined the structure of human PPT in complex with CoA and the ACP domain of human FAS (PDB ID: 2CG5). The serine acceptor residue for the phosphopantetheinyl moiety in ACP was mutated to alanine to facilitate crystallization of a stable ternary complex of PPT, apoACP, and CoA. Thus, as far as we know, this study constitutes the first description of a type I ACP molecule in complex with both CoA and one of its cognate interacting proteins.

The structures of both type II ACPs and peptidyl carrier proteins superimpose onto that of human cytosolic FAS ACP with rmsds generally <3 Å. Whereas the published type I rat ACP structure determined by NMR (1N8L [18]) shows clear differences in the N-terminal loop segments (rmsd >5 Å), a recently deposited rat type I ACP NMR structure (2PNG) is more similar to human cytosolic ACP with rmsd for α-carbons ∼2 Å. As the first structure (1N8L) is indicated as being a preliminary model, the differences with the human ACP in the N-terminal region can be disregarded.

**Figure 3:**
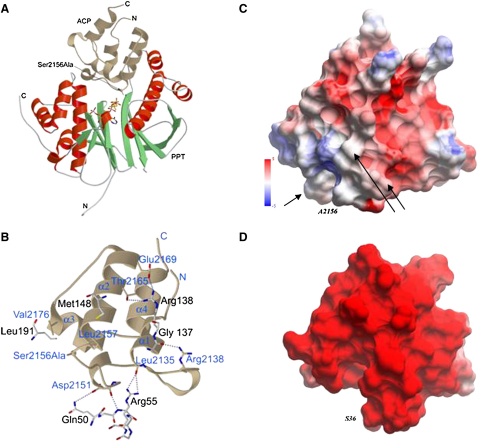
ACP Binding to Human PPT (A) Ribbon model of human PPT (green/red) with ACP (light brown). The mutated Ser→Ala acceptor residue of ACP is indicated in stick representation (arrow). (B) Detailed PPT-ACP interactions. ACP is shown as light brown ribbon model with interacting residue side chains (labeled in blue). PPT residues are depicted as stick models (labeled in black). (C and D) Local electrostatic potential of the α2 (recognition) helices of human ACP (C) and *E. coli* ACP (D), calculated with the REBEL module implemented in the ICM software package [36]. Red color indicates negative charge, blue color positive charges, ranging from −5 to +5 kcal/e.u. charge units. The Ser acceptor positions are depicted at the lower left corner of the ACP molecule. Arrows indicate positions of the hydrophobic recognition patches.

Although sequence identities between different ACP forms are as low as 20%, all ACPs that have been structurally characterized display a four-helix bundle arrangement in which the serine residue targeted for phosphopantetheinylation is located on the surface of the protein at the N terminus of helix-α2 (Figure 3). ACP domains associated with type I fungal FASs deviate slightly from this pattern in that they consist of two four-helix subdomains, the second of which carries the postranslationally added phosphopantetheinyl moiety [16, 19].

The type I human ACP binds in the cleft between the two domains of the PPT and the essential Ser2156Ala points directly toward the β-phosphate of the bound CoA. The interaction between the two proteins takes place on a large interface and is mostly of hydrophobic character. Three hydrophobic patches on the PPT surface interact with the ACP. The first is formed by PPT residues Phe51-Ala54 and interacts with a hydrophobic island formed by ACP residues Ile2137, Val2143, and Leu2152. The second consists of the exposed side chain of Leu191 on the PPT surface and Val2176 on the ACP. Possibly, the hydrophobic pantetheinyl of CoA also interacts with this patch as it is pointing toward this direction, but it cannot be located in the electron density map and therefore has not been modeled. The third hydrophobic interface consists of PPT residues Phe144-Met148, Phe173, and Trp177 interacting with ACP residues Leu2157, Met2158, and Val2160. Elimination of this hydrophobic patch by replacement of all three amino acids with alanine residues decreased the Km for ACP by at least three orders of magnitude, consistent with a role for the hydrophobic patch in recognition by the PPT (data not shown). A few polar interactions that involve hydrogen bonds and salt bridges can be observed (ACP-PPT: Arg2138-Gly137, Asp2151-Gln50, Thr2165-Arg138, Glu2169-Arg138), but binding specificity seems to be determined by shape complementarity rather than through the few specific interactions.

Although these polar interactions are largely mediated by residues located on ACP helix α2, which is categorized for type II ACPs as the “universal recognition helix” [20], the type of recognition clearly is different between type I and II ACPs. In type II ACPs, highly conserved negatively charged residues play a dominant role in mediating binding to their cognate proteins [20–22]. For example, the ability of the group I *E. coli* PPT to catalyze phosphopantetheine transfer to heterologous type II ACPs involved in polyketide synthesis correlates closely with the overall negative charge on the apo-ACP substrates [23], and the complex between the Group I *B. subtilis* PPT and its cognate type II ACP is stabilized predominantly by hydrophilic contacts [11]. However, a comparison of the recognition surfaces of type II (*E. coli*) ACP and type I (human cytosolic FAS) ACPs reveals a considerably lower overall negative charge in the human ACP (Figure 3). Based on analysis of sequence alignments, this feature appears to be a characteristic of the ACP domains associated with both the type I fatty acid and polyketide synthases. So, although the positioning of the human ACP in complex with its cognate PPT is similar to that found in the type II ACP:AcpS complex (PDB ID: 1F80) and the same helix-α2 structural element appears to play a key role in this interaction, hydrophobic residues appear to make a stronger contribution in the type I systems.

Upon binding the ACP, the two domains of PPT rotate around a hinge consisting of residue Glu133, and close slightly (3.9 Å at the furthest point from the hinge, or approximately 10 degrees). This motion is not expected to have a significant impact on catalysis since the conformation of active-site residues remains nearly identical, except for Glu138 that is involved in Mg binding; in the ACP complex, the lack of Mg supposedly lets the Glu138 side chain rotate into a different position. In the PPT-CoA complex, the long C-terminal coil of the enzyme (residues 290–305) is highly ordered due to its participation in crystal packing with two other subunits in the crystal lattice. For the PPT-CoA-ACP complex, the packing within the crystal lattice is different, and this region is no longer visible in the electron density maps, indicating inherent flexibility. On the other hand, the position of the bound CoA is changed only slightly on binding of ACP, and the phosphopantetheine moiety is now disordered, possibly oriented away from the active site to avoid collision with the bound ACP. Structures of PPT with CoA bound show variable position of the pantetheine moiety, suggesting this part of the molecule does not have a constrained conformation [11] (PDB ID: 2jbz). This is also in agreement with the fact that modified CoAs such as acetyl-CoA can act as substrates for PPT. Surprisingly, in the PPT-CoA-ACP complex, no density was found for the Mg that is sequestered in the active center of the PPT-CoA complex. This may be explained by a water molecule that coordinates the Mg in the PPT-CoA structure, which is displaced by the Ala side chain (substituted for the Ser2156) when complexed with ACP. The β-phosphate of CoA also moves closer to the acceptor Ser2156Ala and to the catalytic residue Glu181 (see below). No significant motion can be detected among the side chains of active-site residues (except for Glu181, which is pushed slightly back by the movement of the β-phosphate of CoA), indicating that binding of CoA alone positions them suitably for catalysis.

### CoA and Mg Binding to Human PPT

**Figure 4:**
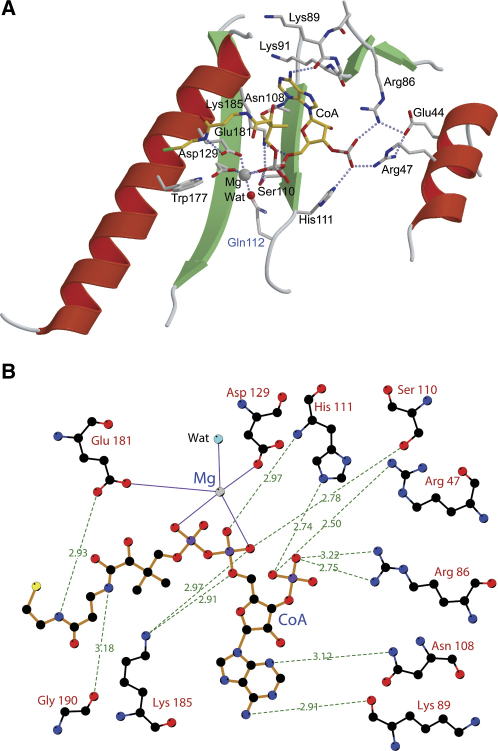
Ligand Binding of Human PPT-CoA Complex (A) Detail of CoA and Mg binding to human PPT. The side chains of residues involved in binding are drawn as sticks and labeled. The hypothetical model for the side chain of Gln112 is represented in light blue (see text for detail). (B) Ligplot [37] representation of CoA and Mg binding with hydrogen bonding interaction distances given in Ångstrom.

In the structure of PPT with Mg^2+^ and CoA (2C43), the ligands are found in the active site formed in a shallow groove at the interface of the two domains (Figure 2A), similar to the binding mode of these ligands determined for bacterial Sfp [13]. CoA is bound in a bent conformation with the pyrophosphate group at the apex of the bend, and clear density is observed for all parts of the molecule including the phosphopantheteinyl moiety (Figure 2B). As observed in other CoA complex structures, the adenine is bound to the adenine ribose in an antiglycosidic torsion angle. The ribose is in a 3′-endo conformation, similar to the conformation in complex with *B. subtilis* Sfp [13]. The 3-phospho-adenosine portion of CoA is tightly bound through a combination of hydrophobic and hydrogen-bonding interactions contributed by residues Lys89, Lys91, Asn108, Arg47, and Arg86 (Figure 4). The latter two basic residues play a key role by forming salt bridges to the oxygen atoms of the 3′-phosphate of the adenine ribose. In Sfp, this role is played by Lys28 and Lys31, but although Lys31 corresponds to Arg47 in PPT, the equivalent residue to Lys28 is in fact Glu44 and, since it is negatively charged, cannot contribute to phosphate binding (Figure 2E). Since Glu44 is within hydrogen-bonding distance to both Arg47 and Arg86, this change may be necessary to stabilize the two highly charged Arg side chains. The residue corresponding to Arg86 is Thr70 in Sfp and, as the shorter side chain suggests, plays no role in CoA binding. In addition, the Nɛ2 nitrogen of the imidazole side chain of His111 binds to the 3′-phosphate group. The α-phosphate group of the pyrophosphate moiety is held in place by interactions with the main-chain amide of His111 and the side-chain hydroxyl of Ser110, while the phosphopantheteinyl group is bound through interactions with side and main chains of residues Glu181, Gly190, Leu191, and Leu195. Magnesium is coordinated by the α and β groups of the CoA pyrophosphate, the carboxylates of Asp129 and Glu181, and one water molecule (Figure 4). Although the side chain of Gln112 is disordered, modeling indicates that it could participate in Mg coordination by substituting its side-chain amide oxygen for the ordered water. A sixth ligand for the Mg is not present in the final crystallographic model; however, there is some residual density that implies this position may be occupied by a poorly ordered water molecule. The metal cation is found in a solvent inaccessible area, shielded by protein and CoA moieties. As found in many other Mg binding proteins [24], PPT has, besides the inner coordination sphere of the ligands described above, a hydrophobic outer shell that is provided by residues Ile130, Met131, Tyr174, and Trp177, thus creating an environment of high hydrophobic contrast.

The cocrystallization experiments with PPT, CoA, Mg, and a Ser2156Ala mutant of human cytosolic-FAS ACP clearly showed that Mg was not present in this structure (PDB ID: 2CG5) (Figure 3), unequivocally demonstrating the nonessential nature of Mg for CoA binding. As described above, the Ala2156 side chain pushes out the water molecule that is bound to Glu181 in the PPT-CoA structure. Elimination of the water causes Glu181 to adopt a different side-chain conformation, which no longer contributes to Mg binding. This water would presumably be replaced by the Ser-OH acceptor group in a productive PPT-CoA-ACP complex. Taken together, this indicates CoA binding is mainly dependent on conserved residues binding to the 3′-adenosine phosphate (Arg47, Arg86, and His111) as well as to the α-phosphate (Lys185, Ser110, and His111) but not on binding of the divalent cation. Protein ligands critically involved in Mg binding are side chains of Glu181 and Asp129 as deduced from the structural and kinetic data.

### Kinetic Analysis of Human PPT

Residues likely involved in Mg and CoA binding were evaluated by determining the effects of site-directed mutagenetic replacements on catalysis. Residues found to be highly conserved in the PPT family comprise in the human enzyme Arg47, Arg86, His111, Gln112, Asp129, Glu181, and Lys185. These residues were replaced as indicated in Table 1.

Kinetic analyses (Table 1) revealed that replacement of Glu181 (with Ala or Gln) or Asp129 (with Ala) has a relatively small effect on K_m_ for CoA but reduces the affinity for Mg^2+^ by 20- to 40-fold and k_cat_ by three orders of magnitude; thus, catalytic efficiency is reduced by four orders of magnitude. These findings, together with the crystallographic data are consistent with a key role for Glu181 and Asp129 side chains in Mg^2+^ binding. Though only partially defined in the electron density maps, the crystallographic analysis also implies that the side chain carbonyl of Gln112 could be involved in Mg^2+^ binding but replacement with Glu had little effect on the K_m_ for Mg^2+^, whereas k_cat_ was decreased by an order of magnitude. In contrast, replacement of Glu181 with Gln elevated the K_m_ for Mg^2+^ ∼20-fold and decreased k_cat_ > 300-fold. Exchange of the Glu and Gln side chains at positions 112 and 181 in the double mutant elevated the K_m_ for Mg^2+^ > 200-fold and reduced k_cat_ ∼3 orders of magnitude, emphasizing the importance of the position of the two side chains for proper coordination of Mg^2+^. Alanine replacement of three residues involved in CoA binding, Arg47, Arg86, or His111, resulted in a lowered affinity for CoA. However, the affinity of these mutants for Mg^2+^ was also lowered, suggesting that binding of CoA facilitates the subsequent binding of Mg^2+^. This inference is supported by the crystallographic data that show that Mg^2+^ is bound to CoA via both the α and β groups of the pyrophosphate and that in the crystal structure of the complex with ACP, CoA is present but Mg^2+^ is not. Importantly, k_cat_ for the two arginine mutants was significantly elevated, suggesting that, in the wild-type enzyme, the rate of release of the 3′,5′-ADP product may be limited by interaction of the two guanidinium moieties with the 3′-phosphate. Replacement of Lys185 has no major effect on affinity for either CoA or Mg but results in a reduction of k_cat_ by three orders of magnitude, consistent with the primary role of this residue in the protonation of the α-phosphate that accompanies pyrophosphate cleavage.

### The Catalytic Mechanism of Human PPT

**Figure 5:**
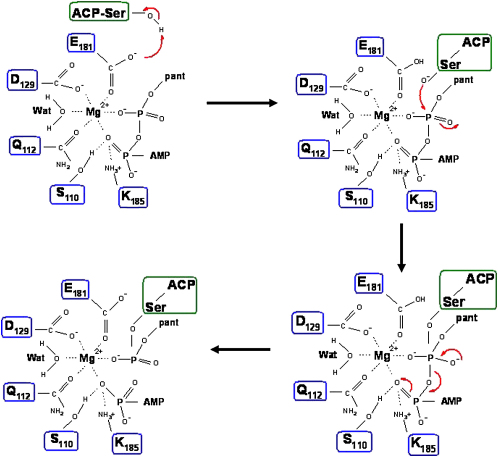
Proposed Reaction Mechanism for Human PPT Residue E181 is the acid/base catalyst that abstracts a proton from Ser2156 of ACP, and cleavage of the pyrophosphate bond is achieved after charge migration and protonation of the α-phosphate likely achieved through Lys185, resulting in dissociation of the products from PPT.

Based on the structural and functional data obtained, we propose the following catalytic mechanism for human PPT (Figure 5): after initial CoA and Mg binding, ACP completes the complex. A proton is abstracted from the ACP-Ser-hydroxyl group by Glu181, facilitated by a helix-dipole effect from the α2 helix on ACP. The resulting ACP-Ser-hydroxylate carries out a nucleophilic attack on the β-phosphate of CoA, and after charge migration over the α-phosphate and protonation by Lys185, the pyrophosphate is cleaved, and the products dissociate from the enzyme. A rate limiting step is likely to be the dissociation of the 3′,5′-ADP product from the enzyme, indicated by the significantly higher V_max_ values obtained for mutants Arg47Ala and Arg86Ala, which are involved in binding this portion of the CoA molecule. Besides the structural data observed in the complexes, the role of Glu181 and Lys185 as key acid/base catalysts is strongly supported by the observed significant loss of activity for the Glu181Gln, Glu181Ala, and Lys185Ala mutant proteins. In addition to being a key catalyst, Lys185 is also involved in binding the α-phosphate, whereas Glu181 does not contribute to CoA but to Mg binding. The function of Asp129 is compatible with a role in Mg coordination (indicated by a loss of activity for the Ala substitution) but not in CoA binding. This proposed mechanism for human and presumably all other eukaryotic group II PPTs is similar to the suggested Sfp mechanism [12] but significantly different from the sequence of catalytic steps outlined for the group I PPT AcpS, where a metal-ion-activated water molecule is proposed to abstract a proton from the acceptor Ser-hydroxyl group [11]. This is further supported by the significantly reduced pyrophosphate cleavage activity for group II PPTs (∼five orders of magnitude) in the absence of an acceptor molecule.

In summary, our study reveals fundamental differences in the active site of the group II human PPT and the group I bacterial PPTs, not only in architecture, but also in the reaction mechanisms. The group I PPTs have a divalent cation coordinated by the CoA α-phosphate, an Asp and a Glu residue, and three water ligands, one of which is proposed to act as a catalytic base [10, 11]. In the group II human PPT, the divalent cation is coordinated by the CoA α- and β-phosphates, as well as two to three protein side chains and a water molecule. Uniquely in the group II human PPT, a protein carboxylate side chain is directly involved in catalysis. Recently, a novel class of anthranilate compounds was reported as lead candidate for bacterial group I PPT targets [25], and screening of these compounds in a PPT binding assay did not indicate significant interactions with the human enzyme (data not shown), consistent with the observed fundamental differences in ligand binding and reaction mechanisms between group I and II PPTs. Finally, hydrophobic interactions appear to play an important role in stabilizing the complex between the group II human PPT and its cognate type I ACP, whereas ionic interactions are more critical in stabilizing complexes between the group I PPTs and their cognate type II ACPs.

## Significance

**Cytosolic FAS has been discussed as a potential target for the development of drugs for the treatment of obesity and cancer** **[26, 27]****. Thus, inhibitors of the human PPT that would block the formation of a functional holo-FAS might also be considered useful in this regard. Surprisingly, however, mammals appear to use a single PPT for the posttranslational modification of the ACP domain of the cytosolic FAS and at least two other proteins: the ACP component of a mitochondrial FAS** **[22, 28–32]** **that is involved in the synthesis of the lipoyl moieties required for the posttranslational modification of several key mitochondrial enzymes** **[33]** **and the aminoadipate semialdehyde dehydrogenase involved in lysine degradation** **[2]****. Therefore, anti-human-PPT agents would be expected to impact lipid synthesis, mitochondrial metabolism and amino acid metabolism and are unlikely to find therapeutic application. Nevertheless, group I prokaryotic PPTs are being investigated as possible targets for the development of new antibacterial agents that could inhibit the production of bacterial lipopolysaccharides and membrane lipids** **[8, 34]****. So it will be important to establish that these agents exhibit high selectivity and do not target the human PPT. Our study reveals fundamental differences in the active site of the group II human PPT and the group I bacterial PPTs, not only in architecture but also in the mechanism of interaction with the CoA, divalent cation, and apoACP. This information could be exploited in the design of inhibitors that target specifically the bacterial group I PPTs.**

## Experimental Procedures

### Cloning, Expression, and Purification of Human PPT and the ACP Domain of FAS

A construct encoding human PPT (template was obtained from the MGC clone collection) was cloned by PCR into a pCOEX1 vector, resulting in an N-terminally His_6_-tagged variant with a TEV protease site. The construct was expressed in the phage-resistant *E. coli* expression strain BL21(DE3)-R3 in TB medium supplemented with 34 μg/ml chloramphenicol. High-level soluble protein production was achieved by IPTG induction (1 mM) at 25°C for 4 hr. Cells were disrupted in a high-pressure homogenizer followed by sonication, nucleic acids were precipitated by addition of polyethyleneimine (0.15%), and the resulting cell lysate was centrifuged for 20 min at 40,000 × g. The clarified supernatant was filtered and then subjected to immobilized metal affinity chromatography (Ni-NTA resin, QIAGEN) and eluted in 250 mM imidazole, 500 mM NaCl, 50 mM HEPES (pH 7.5), 5% glycerol. This was followed by a final gel filtration chromatographic step on a Superdex 200 HiLoad 26/60 column (GE Healthcare, Uppsala, Sweden). Protein was purified in 10 mM HEPES (pH 7.5), containing 2mM TCEP, 5% (w/v) glycerol, and concentrated to 20 mg/ml by using a 30,000 kDa MW cutoff Amicon Ultra concentration device (Millipore, Bedford, MA), resulting in a homogenous and pure protein preparation. The experimentally determined mass by ESI-TOF mass spectrometry (Agilent LC-MSD TOF) was in agreement to the predicted mass value. A construct encoding residues 2117–2205 of the human FAS together with a tev-cleavable N-terminal His6 tag was expressed in *E. coli*, and the apoprotein purified as described previously [8]. The apoACP was used for crystallization without cleavage of the N-terminal tag.

### PPT Mutagenesis

PPT point mutations were generated with QuickChange XL site-directed mutagenesis kit (Stratagene, USA) and wild-type human PPT cDNA expression plasmid as DNA template. Appropriate mutant primers were designed and mutagenesis carried out according to the manufacturer's instructions. Authenticity of all mutants was confirmed by DNA sequencing.

### Expression and Purification of PPT

For activity measurements, WT and mutants were expressed in *E. coli* BL21 (DE3) with TB medium containing 34 μg/ml chloramphenicol in 4 × 500 ml cultures, each in 2 l flasks. Cells were grown at 37°C to a density corresponding to A600nm of 2, transferred to 25°C, and then expression was induced for 4 hr by the addition of 1 mM IPTG. Cells were lysed in 50 mM Tris-HCl (pH 7.5), 500 mM NaCl, 10 mM imidazole, 2 mM mercaptoethanol, 10% glycerol, containing protease inhibitors (leupeptin 5μg/ml, trans-epoxysuccinyl-LGB 10 μM, pepstatin 1 μg/ml, and antitrypsin 5 μg/ml) with a high-pressure microfluidizer, and the extract was centrifuged at 214,000 × g, 90 min. The supernatant was filtered through a 0.45 μ membrane, and the enzyme was purified on a column (6 ml) of NiNTA.

The bound PPT was eluted with 50 mM Tris-HCl (pH 7.5), 500 mM NaCl, 250 mM imidazole, 2 mM mercapto EtOH, 10% glycerol, and dialyzed overnight against 50 mM Tris-HCl (pH 7.5)/1 mM DTT/10% glycerol. Further purification was achieved by anion exchange chromatography on a column (6 ml) of Resource Q (Amersham Pharmacia) equilibrated with 50 mM Tris-HCl (pH 7.5)/1 mM DTT/10% glycerol and eluted with a gradient of NaCl (0–0.25M) in the same buffer. The proteins were dialyzed against 50 mM Tris-HCl (pH 7.5)/0.1 M NaCl/1 mM DTT/10% glycerol, homogeneity was confirmed by SDS-PAGE on 10% acrylamide gels, and protein concentration was determined from A280 nm by using extinction coefficients estimated from A280 nm with the ProParam tool at http://www.expasy.ch/tools/protparam.html.

### PPT Kinetics

Mutant PPT activities were assayed at 37°C in 83 mM Tris-HCl buffer (pH 7) containing 100 μM apo-ACP, MgCl_2_ and [1-^14^C]acetyl-CoA; final volume was 30 μl. The specific radioactivity of the [1-^14^C]acetyl-CoA substrate, the amount of enzyme used, and the duration of the incubation were varied, according to the activity of the particular mutant, to provide sufficient yield of radiolabeled product and ensure that steady-state conditions always prevailed. Reactions were stopped by the addition of trichloroacetic acid (final concentration, 10%); bovine serum albumin (0.4 mg) was added as coprecipitant, and the precipitate containing [1-^14^C]acetyl-S-ACP was washed three times with trichloroacetic acid, dissolved in 330 μl of 6 M guanidine hydrochloride and assayed for radioactivity by liquid scintillation spectrometry. One unit of activity corresponds to 1 nmol product formed per min. Kinetic parameters are the mean ± SD values from six different methods, derived with EnzymeKinetics (Trinity Software). CoA pyrophosphatase activity was assayed in the absence of an ACP acceptor. Unreacted CoA was derivatized with DTNB and separated from the 3′,5′-ADP product by anion exchange chromatography on a HiTrap Q HR column eluted with a gradient of NaCl, 0–1 M in 20 mM BisTris (pH 7).

### Crystallization and Structure Determination

Initial crystals were obtained from several coarse screen conditions that contained around 20% PEG, 0.20 M salt and had slightly acidic pH. These were further optimized, and conditions were sorted based on diffraction properties. Eventually, long rod-like crystals with average dimensions 0.1 × 0.1 × 0.6 mm^3^ could be obtained from 14% PEG3350 and 0.05 M H_3_Cit/Na_3_Cit (pH 5.7). Since it was established that no suitable molecular replacement model exists and crystals from selenomethionine-labeled protein could not be obtained, a crystal was soaked in a cryoprotectant solution consisting of the mother liquor, 20% PEG400 and 0.5 M KBr, and flash frozen by plunging into liquid nitrogen. Datasets were collected at the PXII beamline at the Swiss Light Source by using 0.9198 Å radiation (peak wavelength determined from a fluorescence scan). Data were processed with XDS, and different scans were scaled together with XSCALE. Data collection and refinement statistics are given in Table 2.

Strong anomalous signal was obtained, and bromide atoms were located with SHELXD. After phase calculation and density modification by SHELXE, ARP/wARP was started that resulted in an initial trace, which was completed manually and recycled for ARP/wARP. After several cycles, a virtually complete trace could be obtained. Refinement was completed with refmac5.

Crystals of the PPT-CoA complex were obtained by cocrystallizing the protein with 5 mM CoA in the presence of 20 mM MgCl_2_. Initial screening was repeated and diffraction quality crystal were obtained from 2.0 M NaCl and 10% PEG6000. PPT was also cocrystallized with equimolar amount of Ser2156Ala ACP, in the presence of 2.5 mM coenzyme A and 12 mM MgCl_2_. Trigonal pyramids could be obtained from 0.4 M NH_4_H_2_PO_4_. Data for both complexes were collected on a Rigaku FR-E rotating anode equipped with Osmic-HR multilayer optics and a Rigaku HTC image plate detector. Datasets were processed with XDS, and the structures were solved with molecular replacement by using the apoprotein as a search model. For the ACP complex, ACP was built manually into the electron density. The structures were refined with refmac5.

Please note that the sequence numbering between the PDB files and the structure discussed in this text is offset by +10.

## Figures and Tables

**Table 1:** Kinetic Properties of Human PPT and Selected Active-Site Mutants

PPT	Varied Ligand	K_m_ (mM)	k_cat_ (min^−1^)	k_cat/_K_m_ (min^−1^ mM^−1^)
WT	Mg^2+^	0.44 ± 0.04	14.4 ± 0.4	32.7
D129A		4.6 ± 0.3	0.0042 ± 0.0005	0.001
H111A		34.5 ± 5.6	7.3 ± 0.6	0.21
R47A		3.3 ± 1.0	48.0 ± 6.6	14.5
R86A		15 ± 3	72.0 ± 5.9	4.8
K185A		1.1 ± 0.1	0.018 ± 0.0004	0.016
E181A		18.3 ± 0.7	0.023 ± 0.0007	0.001
E181Q		7.6 ± 4.6	0.039 ± 0.005	0.005
Q112E		0.75 ± 0.24	1.32 ± 0.21	1.76
E181Q, Q112E		104.1 ± 51	0.019 ± 0.004	0.0002
WT	C_2_-CoA	0.0249 ± 0.003	14.4 ± 0.73	578
D129A		0.142 ± 0.035	0.0059 ± 0.0001	0.04
H111A		0.095 ± 0.033	6.1 ± 1.3	64.2
R47A		0.076 ± 0.0038	34.0 ± 1.2	447
R86A		0.399 ± 0.041	58.4 ± 3.8	146
K185A		0.0247 ± 0.0036	0.015 ± 0.0004	0.6
E181A		0.0454 ± 0.0038	0.043 ± 0.0015	0.9
E181Q		0.081 ± 0.0013	0.044 ± 0.0026	0.5
Q112E		0.047 ± 0.023	2.2 ± 0.73	46.8
E181Q, Q112E		0.093 ± 0.001	0.024 ± 0.004	0.2

Kinetic parameters for Mg^2+^ were determined at saturating concentrations of acetyl-CoA and vice versa.

**Table 2:** Data Collection and Refinement Statistics

	apoPPT	PPT + CoA	PPT + ACP + CoA
PDB ID	2BYD	2C43	2CG5
Space group	*P*2_1_2_1_2_1_	*P*2_1_2_1_2_1_	*P*3_2_21
Unit cell	a = 63.78 Å, b = 69.95 Å, c = 71.24 Å	a = 65.59 Å, b = 68.96 Å, c = 70.75 Å	a = b = 69.36 Å, c = 184.7 Å
No. of unique reflection	21391	19791	14864
Resolution	47.5–2.00 (2.10–2.00)	32.8–1.93 (2.02–1.93)	34.7–2.70 (2.80–2.70)
Completeness (%)	96.4 (77.6)	80.3 (29.2)	99.9 (100)
Redundancy	6.6 (4.0)	5.0 (0.7)	10.4 (10.6)
I/σ	11.8 (2.23)	16.5 (3.59)	17.9 (3.35)
R_int_	0.128 (0.578)	0.084 (0.245)	0.090 (0.582)
R_cryst_/R_free_	0.176/0.242	0.161/0.219	0.195/0.247
Rmsd bond length (Å)	0.010	0.017	0.013
Rmsd bond angle (°)	1.28	1.66	1.50
No. protein atoms	2315	2276	2638
No. hetero atoms, ions	9	50	46
No. water molecules	189	180	24

Highest resolution shell indicated in parentheses.

## Accession Numbers

Models of the PPT apostructure, PPT-CoA complex, and PPT-CoA-ACP ternary complex were deposited into the Protein Data Bank under accession numbers 2BYD, 2C43, and 2CG5, respectively.
